# Leukocytes in Critical Patients With Asthma Exacerbation

**DOI:** 10.7759/cureus.20520

**Published:** 2021-12-19

**Authors:** Hussein Rabah, Ahmad Itani, Michel Chalhoub

**Affiliations:** 1 Internal Medicine, Staten Island University Hospital, New York, USA; 2 Pulmonary and Critical Care Medicine, Staten Island University Hospital, New York, USA

**Keywords:** innate immune system, adaptive immunity, medical intensive care unit (micu), leukocytes, asthma exacerbation

## Abstract

Background

Asthma exacerbations, defined as acute or subacute progressive worsening of airway spasm, are a significant cause of disease morbidity. Risk factors for exacerbation include sex, age, race, socioeconomic status, baseline lung function, smoking history, and exposure to respiratory viruses. It is believed that white cells play an essential role in the pathogenesis of such attacks; however, the current understanding of the relationship between cell lines during an asthma attack is minimal.

Methods

This report represents a retrospective study for patients admitted to ICU for asthma exacerbations. The Medical Information Mart for Intensive Care iii (MIMIC iii) version 1.4 database was used to identify patients admitted for asthma exacerbations. The demographics, laboratory data gathered in addition, to clinical variables and outcomes were determined.

Results

The length of stay increased with the increase in WBC (p = 0.001). Intubated patients had an increased white blood cell (WBC) count when compared with non-intubated patients (p-value 0.009). In addition, patients with normal basophil counts on presentation were less likely to need intubation than those presenting with low basophils (p-value 0.015, adjusted odds ratio = 0.074, CI [0.009-0.620]) and those presenting with basophilia (p-value 0.001, adjusted odds ratio = 0.025, CI [0.003-0.225]). Furthermore, prolonged intubation (for three days or more) was positively correlated with eosinophil counts. On the other hand, there was no statistically significant association between the length of ICU and the patient's age, smoking status, or gender (p-values 0.611; 0.761; and 0.201, respectively).

Conclusion

Asthma exacerbation is a disease of heterogeneous pathophysiology. The leukocyte count is associated with the length of stay and the need for mechanical ventilation.

## Introduction

Asthma is a chronic inflammatory disease of the airways. It is characterized by episodic airflow restriction and airway narrowing, representing an excessive airway response to various stimuli. Its pathophysiology involves genetic, epigenetic, and environmental factors leading to persistent inflammatory, functional, and structural airway changes. Exacerbations, defined as acute or subacute progressive worsening of asthma, are a significant cause of disease morbidity and are associated with a decline in pulmonary function [1]. Risk factors for exacerbation include sex, age, race, socioeconomic status, baseline lung function, smoking history, and exposure to respiratory viruses [2].

It has been found that besides Th2 cells, other inflammatory cells play a role in the pathogenesis of asthma [3]. Studies of the airway during asthma exacerbations suggest an inflammatory infiltrate with a mixture of neutrophils, eosinophils, and mast cells [4]. Such findings suggest that the pathogenesis of asthma exacerbations and that of chronic asthma are different.

This study aims to compare the concentration of white blood cells in peripheral blood among patients admitted to the intensive care unit due to asthma exacerbations, as well as their respective inflammatory cell ratios, and describe their association with clinical outcomes.

## Materials and methods

Study design

This report represents a retrospective study for patients admitted to the ICU for asthma exacerbations. The Medical Information Mart for Intensive Care iii (MIMIC iii) version 1.4 database was used to identify patients admitted for asthma exacerbations using the International Classification of Diseases, 9th Revision: 493.xx [5]. The demographics, including age, gender, and race, were determined for each patient. Laboratory data gathered include white blood cell (WBC) counts, neutrophil, eosinophil, monocyte, basophil, and monocyte counts on admission. In addition, the length of stay (in days), oxygen requirements, oxygen delivery method (nasal cannula, face mask, non-rebreather mask [NRB], non-invasive ventilation [NIV]), and the need for invasive mechanical ventilation (IMV) were determined. After reviewing the study proposal, the IRB concluded that this study is not human subject research due to the de-identification of the patients.

Study population

Patients were identified using the following inclusion criteria: asthma exacerbation medical code (International Classification of Diseases, 9th Revision: 493.xx) and age 18 years or older [5]. Patients with chronic obstructive lung disease or other pulmonary diseases, including malignancy or pulmonary metastasis, and patients with congestive heart failure were excluded.

Statistical analysis

Data analysis was performed using SPSS version 24.0 (Armonk, NY: IBM Corp). Continuous and categorical variables were presented as mean ± standard deviation and frequency/percentages, respectively. Lab values were evaluated as continuous, ranges, and ratios. Normality plots were used to evaluate the distribution of continuous variables. Fischer's exact test, independent sample T-test, and ANOVA test were used to evaluate bivariate relationships. Linear and binary logistic regression models were fitted to evaluate the factors of ICU stay and intubation, respectively. To achieve normality of error terms, the ICU stay was square-rooted. Tests were interpreted at a significance level alpha = 0.05.

## Results

Patients

In total, data from 120 patients were included in the study. They were between 19 and 89 years old. The mean age at the time of admission was 45.5 ± 16.3 years. Overall, 88 (73.3%) of the participants were females, whereas 32 (26.7%) were males. In terms of race, 50 patients (41.7%) were Black, 46 (38.3%) were White, 11 (9.2%) were Hispanic, 3 (2.5%) were Asian, and the remaining patients were not categorized. Of the 120 patients, 77 (64.2%) were nonsmokers, and 42 (35%) were smokers. There were 65 (54.2%) who had a food or drug allergy, and 55 (45.8%) who had no history of allergy (Table 1).

**Table 1:** Characteristics of patients admitted to the intensive care unit for asthma exacerbation.

		Frequency	Percent
Gender	Females	88	73.3
	Males	32	26.7
	Total	120	100.0
Race	African American	50	41.7
	White	46	38.3
	Hispanic	11	9.2
	Asian	3	2.5
	Other	10	8.3
	Total	120	100.0
Smoking	Yes	42	35.0
	No	77	64.2
	Total	119	99.2
Allergy	Yes	65	54.2
	No	55	45.8
	Total	120	100.0

Oxygen requirements

Oxygen requirements during the ICU stay were as follows: 38 (31.7%) required no supplemental oxygen, 38 (31.7%) required oxygen therapy via a nasal cannula, 4 (3.3%) required a face mask, 10 (8.3%) required an NRM, and 11 (9.2%) required therapy with NIV/Bipap. IMV was needed for 19 (15.8%) of the patients, and the mean intubation period was 0.6 ± 2 days. The mean ICU stay was 2.6 ± 2.9 days (Tables 2-3).

**Table 2:** Oxygen requirements for patients admitted to the intensive care unit for asthma exacerbation. NIV: non-invasive ventilation

		Frequency	Percentage
Non-intubated	Room air	38	31.7
	Nasal cannula	38	31.7
	Face mask	4	3.3
	Non-rebreather	10	8.3
	Positive NIV	11	9.2
Intubated		19	15.8
Total		120	100

**Table 3:** The ages, duration of ICU stays, and intubation periods in patients admitted to the ICU for asthma exacerbations.

	Age	ICU stay	Intubation days
Mean	45.57	2.661	0.654
Median	44.50	1.600	
Std. deviation	16.340	2.9895	2.0477

Cell counts and their ratios

**Figure 1:**
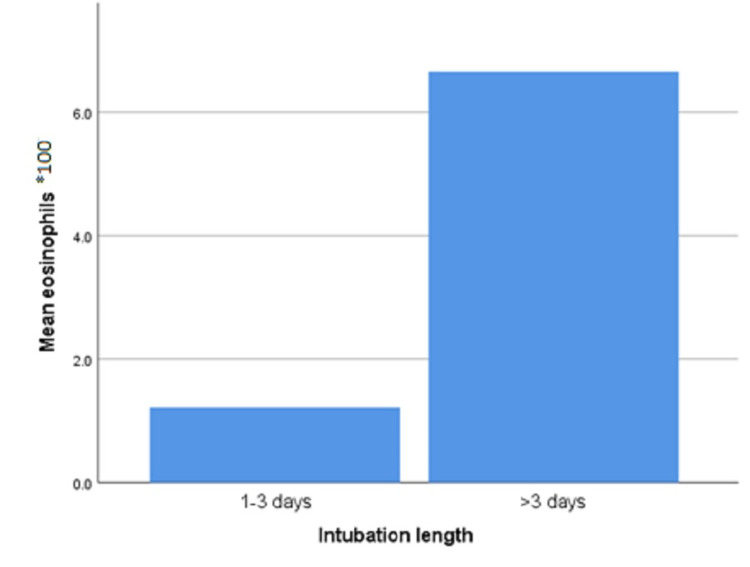
Mean eosinophil count and the length of mechanical ventilation among intubated intensive care unit patients admitted for acute asthma exacerbation.

Intubated patients had an increased WBC count compared with non-intubated patients (p-value 0.009). In addition, prolonged intubation (for three or more days) was positively correlated with eosinophil counts (p-value 0.016; Figure 1) and monocyte to lymphocyte ratio (MLR; p-value 0.001).

The basophil count was a predictor of the need for intubation. Patients with normal basophil counts (between 25 and 100 cells/mm^3^) on presentation were less likely to need intubation than those presenting with low basophils (p = 0.015, adjusted odds ratio = 0.074, CI [0.009-0.620]) and those presenting with basophilia (more than 100 cells/mm^3^; p = 0.001, adjusted odds ratio = 0.025, CI [0.003-0.225]) (Table 4).

**Table 4:** Peripheral basophil cell counts among intubated and non-intubated patients admitted to the ICU for severe asthma exacerbation.

		Basophil count			Total
		Low	Normal	Elevated	
Non-intubated	Frequency	32	54	11	97
	Percentage	33%	55.6%	11.4%	100%
Intubated	Frequency	8	1	8	17
	Percentage	47%	5.8%	47%	100%
Total	Frequency	40	55	19	114

Most of the different cell counts and their ratios did not correlate with the oxygen requirements (neutrophils (N) p-value 0.178, lymphocytes (L) p-value 0.504, eosinophils (E) p-value 0.182, basophils (B) p-value 0.079, monocytes (M) p-value 0.686, N/L p-value 0.398, E/N p-value 0.2, and E/B p-value 0.2). No differences among races were noted concerning intubation (p-value 0.491), stay (p-value 0.563), and WBC counts (p-value 0.278).

Regression analysis

**Figure 2:**
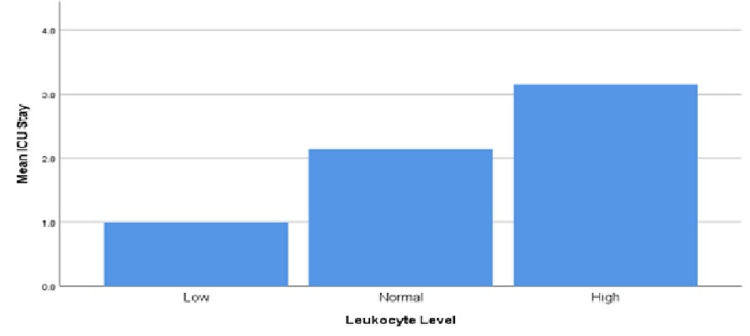
Leukocyte count and intensive care unit stay in patients admitted for severe asthma exacerbation.

The length of ICU stay was not affected by the patient's age (p-value 0.611), smoking status (p-value 0.761), or gender (p-value 0.201). On the contrary, the length of stay increased with the increase in WBC, which was defined as a WBC of more than 11,000 cells/mm^3^ (p-value 0.001; Table 5 and Figure 2).

**Table 5:** Regression analysis showing the correlation between ICU stay and different variables.

	Significance	95.0% confidence interval
Smoker	0.761	−0.301 to 0.22
Sex	0.201	−0.46 to 0.098
Age	0.611	−0.006 to 0.01
WBC	0.001	0.017–0.062

## Discussion

Asthma exacerbations are an exaggerated airway response to various environmental stimuli and are the primary cause of hospitalization in asthmatic patients. Those episodes can be severe and require medical intervention. Etiologies that trigger asthma exacerbation include respiratory infection, cigarette smoke, allergens, and pollutants [6].

The pathogenesis of such attacks is complex; however, evidence suggests that the interaction between the innate immune response and the adaptive immunity microenvironment in the lung causes immune dysregulation and triggers an acute asthma attack [7]. The innate immune system consists of components that do not require prior exposure to an antigen in order to initiate a rapid immune response [8]. On the contrary, the adaptive immune response is more antigen-specific and long-lasting. Despite being classified as two subtypes of the immune system, those two immune responses are well integrated as a single defense mechanism. Adaptive immune responses are regulated through cytokines produced by the innate system [9], and therefore, the innate immune system appears to play a crucial role in determining the type of adaptive immune response.

The innate immune system interacts with antigens through a group of receptors called toll-like receptors (TLR). When a ligand is bound to the receptor, the latter binds adapter proteins that initiate signaling cascades, causing cytokine release and cell recruitment [10]. Some studies have found that activation of toll-like receptors is associated with increased allergic inflammation [11].

As a part of the innate immune system, bronchial epithelial cells express TLR, CD40, and intercellular cell adhesion molecule-1 (ICAM-1) in addition to major histocompatibility complex (MHC), allowing them to present antigens to T cells and induce an inflammatory response in the presence of interferon-gamma (IFN-γ) [12]. On the other hand, antigen-presenting cells (APC) recognize and bind their target antigen using the TLR, as well as other processes such as pinocytosis and endocytosis, which are expressed on the cellular surface. The APC then processes the antigen and displays it on the surface using the MHC. Naive T cell binds the displayed antigen using their antigen-specific T cell receptor (TCR), and are costimulated through additional receptors. The CD4+ lymphocyte then differentiates into one of the three major subsets of T helper (Th) cells (Th1, Th2, and Th17). Th1 cells primarily secrete IFN-γ and interleukin (IL) 2, Th2 cells secrete specific IL proteins, including IL-4, IL-5, and IL-13, and Th17 cells secrete cytokines such as IL-17A, IL-17F, and IL-22 [13].

Th1 cells are mainly involved in intracellular pathogens elimination and are associated with autoimmunity. The primary role of IFN-γ is to enhance phagocytic abilities by activating phagocytes [14], while IL2 promotes the proliferation of CD8+T and memory cells [15,16].

Th2 cells play a critical role in fighting extracellular parasites and the initiation and persistence of asthma and other allergic diseases. IL4 is a principal cytokine involved in allergic reactions. It causes IgE secretion by B cells that bind to mast cells and basophils, leading to their degranulation and release of several active metabolites, consequently triggering an allergic reaction [17]. IL4 also induces the increase of granulocyte-macrophage colony-stimulating factor (GM-CSF) and vascular cell adhesion molecule-1 (VCAM-I) [18]. IL5 is a potent cytokine that induced the maturation and migration of eosinophils. On the other hand, IL9 activates several cells, including mast cells, B cells, eosinophils, neutrophils, and airway epithelial cells. In addition, it induces airway mucus and chemoattractant secretion [19].

Th17 cells are involved in immunity against extracellular microbes and have a role in the pathogenesis of autoimmune diseases [20]. IL17 induces proinflammatory cytokines, including IL6, IL1, and TNF-α, in addition to chemokines needed for inflammatory cells' chemotaxis to sites of inflammation [21].

Another cell that presents a crucial regulatory element during an inflammatory response is the T regulatory (Treg) cell. After the clearance of pathogens, those cells negatively regulate the immune response to maintain immunologic tolerance to self and prevent immunopathology [22]. IL10, TGF-β, and IL35 are their primary cytokines. IL10 is an effective inhibitory cytokine that limits tissue damage by suppressing the inflammatory response [23], and it represses, in addition to TGF-β, IgE production [24].

For many years, it was considered that asthma is a Th2-type disease, where the inflammatory reaction driven by Th2 cytokines predominates over the Th1 cytokines. However, growing evidence suggests that both Th1 and Th2 responses have a role in asthma, and the disease represents a heterogenous result of multiple pathways rather than a homogeneous pathology [25]. In addition, it is proposed that neutrophils also play a role in asthma pathophysiology [26].

Such complex biological interactions between inflammatory pathways are expressed as variable clinical presentations. Eosinophilic asthma (EA) is a phenotype characterized by an elevated peripheral eosinophil count. It is classified as allergic or non-allergic asthma. The allergic subtype is IgE-mediated and has an early onset in life. Patients diagnosed with this subtype often have an atopic background.

On the other hand, the non-allergic subtype is characterized by late-onset and non-elevated IgE levels. Patients with no peripheral eosinophilia are considered to have non-eosinophilic asthma (NEA). Such patients usually have severe diseases and are steroid-resistant [27]. In this study, the numbers of patients admitted to the ICU for asthma exacerbations were almost equal for each phenotype, where 51% had EA and 49% had an NEA phenotype, showing that asthma is a heterogenous pathology rather than a single entity disease.

There is mounting evidence that leukocytosis predicts survival in different medical conditions, such as cancers [28]. Jo et al. concluded that elevated white counts are associated with poor outcomes in patients with pulmonary embolism [29]. Dacey et al. found that leukocytosis was an independent predictor of mortality and poor outcome in coronary artery bypass graft (CABG) patients [30]. Furthermore, leukocytosis is a part of the systemic inflammatory response syndrome (SIRS), defined as an excessive immune response against a pathogenic factor mediated by an increased level of cytokines, causing organ damage [31]. The inflammatory response driven by the innate and adaptive immune systems during an acute asthma attack leads to an increase in the leukocyte count mediated by the chemokines and cytokines released. Therefore, leukocytosis may indirectly estimate the degree of inflammation during an exacerbation, reflecting the disease's severity. In this study, an increase in leukocyte count was associated with a longer ICU stay (p 0.001, CI [0.017-0.062]; Figure 2 and Table 5). Further analysis did not show any significant association between the length of ICU stay and any type of leukocytes or their ratios.

Leukocytes and intubation

Patients with severe asthma exacerbations usually respond to first and second-line therapies. However, about 30% of asthmatic patients admitted to the ICU do not respond to such therapies and require mechanical ventilation [32]. The decision to initiate mechanical ventilation should be based on the severity of airflow limitation (peak expiratory flow), the degree of respiratory distress (tachypnea, inability to talk in complete sentences, accessory muscle use, intercostal retractions, fatigue), hypoxemia, hypercapnia, and response to therapy [33].

In this study, 19 patients (15.8%) were intubated for a mean duration of 0.6 days. Patients with leukocytosis on admission were more likely to require mechanical ventilation (p-value 0.009).

During an allergic reaction, IgE binds to basophils, promoting their degranulation and release of histamines and leukotrienes, which contributes to airway smooth muscle contraction and anaphylaxis. In addition, basophils can be activated by cytokines. IL-3 induces basophils' development and activation and promotes the release of IL-4 and IL-6 from basophils in an IgE-independent manner [34]. Studies also suggest that basophils might interact with dendritic cells during airway inflammation [35]. Although the participation of basophils in the pathogenesis of asthma in humans is not well understood, studies have shown that basophil infiltration is increased in post-mortem lung tissue of patients who have died from severe asthma as well as in bronchial biopsies of patients with asthma [36,37]. In our study, patients who presented with normal basophil counts were less likely to need mechanical ventilation compared to patients with basopenia or basophilia (p = 0.015, adjusted odds ratio = 0.074, CI [0.009-0.620] and p = 0.001, adjusted odds ratio = 0.025, CI [0.003-0.225], respectively; Table 4), thereby showing that the dysregulation of basophils is associated with more severe asthma.

On the other hand, studies have reported that blood eosinophil count is an important factor in predicting asthma exacerbation [38]. Those cells release several mediators, such as major basic protein (MBP), radical oxygen species, cytokines, such as granulocyte-macrophage colony-stimulating factor (GM-CSF), and interleukin (IL)-8 [39,40]. Their granule products mediate epithelial cell damage and induce an acute asthma attack [41]. Furthermore, eosinophils contribute to airway remodeling and fibrosis via the release of transforming growth factor (TGF)-β [42]. In our study, prolonged intubation, defined as mechanical ventilation for three or more days, was positively correlated with peripheral eosinophil counts (p-value 0.016; Figure 1). Therefore, eosinophilia might play a role as a predictor of severe asthma exacerbations.

Bronchial macrophages are primary sources of critical proinflammatory cytokines in asthmatic patients, including TNF-α, IL-1β, IL-6, and IL-8 [43,44]. The production of IL-1β and IL-6 by asthmatic alveolar macrophages can enhance IL-5 production by CD4+ T-cells, potentially intensifying eosinophilic inflammation in the airways. In addition, evidence suggests that macrophages secrete IL-17 which promotes neutrophil inflammation during inflammation related to asthma [45]. Thus macrophages can interact and influence lymphocytes in various ways.

The MLR has been demonstrated as a marker in several medical field studies. It was reported as an independent prognostic factor for patients with advanced gastric cancer and hepatocellular carcinoma [46,47]. In the current study, prolonged ventilation was positively correlated with MLR (p-value 0.001). Those correlations could be explained by monocyte recruitment to the site of inflammation, differentiation into macrophages, and their interaction with lymphocytes and other immune cells during an inflammatory response.

Asthma, gender, and race

Asthma is more severe in young boys; however, there is a gender switch at puberty, where the disease becomes more severe in females [48]. Progesterone inhibits the beat frequency of cilia, which may affect the mucociliary clearance during the menstrual cycle [49]. In a series of 1,261 children and adolescents with moderate to severe asthma, IgE levels were higher among boys aged 6-17 years than among girls, but girls had higher IgE levels during puberty (12-14 years). A higher IgE level was associated with more symptoms triggered by external stimulants and was associated with a lower FEV1/FVC ratio [50].

In this study, 73.3% of the patients admitted to the ICU were females. However, gender was not associated with the length of stay, oxygen requirements, or the need for intubation (Tables 1, 5).

Several studies have demonstrated racial differences in asthma-related morbidity. African Americans have a more significant risk of uncontrolled asthma, a lower likelihood of response to treatment, lower lung function, and a higher risk of asthma exacerbations [51]. Socioeconomic factors have been independently linked with increased exacerbation risk; however, racial variations in exacerbation risk are still observed when such risk factors are eliminated [52]. In our study, most of the patients admitted to the ICU were black (41.7%), followed by white and Hispanic. However, no association was found between race and LOS, oxygen requirements, or mechanical ventilation.

## Conclusions

Asthma exacerbation is a heterogeneous pathology rather than a disease of a single entity. A peripheral white cell count has a role in predicting the length of hospital stay and the need for mechanical ventilation, where an increased leukocyte count is associated with invasive mechanical ventilation and an increased intensive care unit stay. In addition, patients with normal basophil counts are less likely to be intubated. Such results might help predict the outcomes of patients admitted to the intensive care unit with acute asthma exacerbation.
